# Differences in Characteristics and Outcome of Patients with Penetrating Injuries in the USA and the Netherlands: A Multi-institutional Comparison

**DOI:** 10.1007/s00268-018-4669-8

**Published:** 2018-05-21

**Authors:** Suzan Dijkink, Pieta Krijnen, Aglaia Hage, Gwendolyn M. Van der Wilden, George Kasotakis, Dennis den Hartog, Ali Salim, J. Carel Goslings, Frank W. Bloemers, Steven J. Rhemrev, David R. King, George C. Velmahos, Inger B. Schipper

**Affiliations:** 1 Department of Trauma Surgery, Leiden University Medical Center, Leiden, The Netherlands; 2 Division of Trauma, Acute Care Surgery and Surgical Critical Care, Department of Surgery, Boston University School of Medicine, Boston, MA USA; 3 Trauma Research Unit, Department of Surgery, Erasmus MC, University Medical Center Rotterdam, Rotterdam, The Netherlands; 4 Division of Trauma, Burn and Surgical Critical Care and Emergency General Surgery, Brigham and Women’s Hospital, Boston, MA USA; 5 Department of Trauma Surgery, Academic Medical Center, Amsterdam, The Netherlands; 6 Present Address: Department of Surgery, Onze Lieve Vrouwe Gasthuis, Amsterdam, The Netherlands; 7 Department of Trauma Surgery, Vrije Universiteit Medical Center, Amsterdam, The Netherlands; 8 Department of Trauma Surgery, Haaglanden Medical Center Westeinde, The Hague, The Netherlands; 9 Division of Trauma, Emergency Surgery and Surgical Critical Care, Massachusetts General Hospital, Boston, USA

## Abstract

**Introduction:**

The incidence and nature of penetrating injuries differ between countries. The aim of this study was to analyze characteristics and clinical outcomes of patients with penetrating injuries treated at urban Level-1 trauma centers in the USA (USTC) and the Netherlands (NLTC).

**Methods:**

In this retrospective cohort study, 1331 adult patients (470 from five NLTC and 861 from three USTC) with truncal penetrating injuries admitted between July 2011 and December 2014 were included. In-hospital mortality was the primary outcome. Outcome comparisons were adjusted for differences in population characteristics in multivariable analyses.

**Results:**

In USTC, gunshot wound injuries (36.1 vs. 17.4%, *p* < 0.001) and assaults were more frequent (91.2 vs. 77.7%, *p* < 0.001). ISS was higher in USTC, but the Revised Trauma Score (RTS) was comparable. In-hospital mortality was similar (5.0 vs. 3.6% in NLTC, *p* = 0.25). The adjusted odds ratio for mortality in USTC compared to NLTC was 0.95 (95% confidence interval 0.35–2.54). Hospital stay length of stay was shorter in USTC (difference 0.17 days, 95% CI −0.29 to −0.05, *p* = 0.005), ICU admission rate was comparable (OR 0.96, 95% CI 0.71–1.31, *p* = 0.80), and ICU length of stay was longer in USTC (difference of 0.39 days, 95% CI 0.18–0.60, *p* < 0.0001). More USTC patients were discharged to home (86.9 vs. 80.6%, *p* < 0.001). Readmission rates were similar (5.6 vs. 3.8%, *p* = 0.17).

**Conclusion:**

Despite the higher incidence of penetrating trauma, particularly firearm-related injuries, and higher hospital volumes in the USTC compared to the NLTC, the in-hospital mortality was similar. In this study, outcome of care was not significantly influenced by differences in incidence of firearm-related injuries.

## Introduction

Worldwide, traumatic injuries are an important cause of death and disability, especially under 45 years of age [1]. In most developed countries, blunt trauma is responsible for the majority of the trauma burden, while roughly 15% of all injuries are caused by penetrating trauma [2]. Despite the lower incidence, penetrating trauma is a considerable health burden leading to premature mortality, permanent disability and psychological problems [3, 4].

The incidence and nature of penetrating injuries differ between countries. In the USA and South Africa, urban epidemics of penetrating injuries are seen, with penetrating injuries being responsible for 20–45% and up to 60–80% of all injuries, respectively [2, 5]. In European countries, the incidence of penetrating trauma is low; for instance, 3–4% of all injuries in the Netherlands are penetrating, and in Switzerland, only 0.2% of all emergency department visits are penetrating injuries [3, 6, 7]. However, in the Netherlands, 70% of the fatal violent incidents penetrating injuries were seen [3]. Besides the varying incidence, differences in penetrating trauma mechanism are also reported. In European countries, stab wounds represent the majority of penetrating injury, whereas in the USA a considerable proportion of penetrating trauma are gunshot wounds. The overall firearm-related mortality rate is roughly six times higher in the USA compared to European countries [3, 7–13].

Both the primary assessment and treatment of patients with penetrating injuries are often highly complex and require a multidisciplinary team. Similar to the American situation, regionalized inclusive trauma systems are implemented in the Netherlands with dedicated Level-1 trauma centers providing 24/7 comprehensive trauma care [14, 15]. However, differences in clinical routine and experience with penetrating injuries may exist between these countries due to the low incidence of penetrating trauma in the Netherlands, potentially affecting the clinical outcome.

The goal of this study was to compare the demographics, trauma mechanism, injury characteristics and outcomes of patients with truncal penetrating injuries treated in urban Level-1 trauma centers in the USA and the Netherlands. We aimed to gain insight into differences in care to identify factors that may influence patient outcome.

## Materials and methods

### Trauma centers

This multi-institutional retrospective cohort study was performed at five Level-1 trauma centers in the Netherlands (Netherlands trauma center (NLTC): Academic Medical Center, Erasmus Medical Center, Vrije Universiteit Medical Center, Haaglanden Medical Center and Leiden University Medical Center) and three Level-1 trauma centers in Boston, USA (US trauma center (USTC): Boston Medical Center, Brigham and Women’s Hospital, and Massachusetts General Hospital). These NLTC and USTC are all located in urban areas with comparable population densities (4200/km^2^ in Boston versus 5000/km^2^ in the Amsterdam–Leiden–Rotterdam region) [16, 17] and comparable violent crime rates (390 and 360/100.000 in Massachusetts and the Dutch region, respectively) [18, 19].

### Patients and data collection

Eligible patients were identified in the trauma registries of the participating centers. All patients over 15 years of age who had been admitted to the NLTC or USTC with truncal penetrating injuries, i.e., penetrating injuries to the neck, thorax, abdomen, back or inguinal area, between July 1, 2011, and December 31, 2014, were included. Patients with isolated penetrating injuries to the head or the extremities (i.e., without truncal penetrating injuries) were excluded. Patients who were managed at another hospital before arriving at the participating hospital or were transferred to another hospital after initial treatment in participating hospital were excluded. Also, patients who died before arrival or arrived more than 48 h after trauma at the emergency department were excluded. Institutional review board permission was obtained from all participating centers.

### Data

Demographic data and injury data, defined according to the Abbreviated Injury Score (AIS, updated 1998) [20], Injury Severity Score (ISS) [21], vital signs and Revised Trauma Score (RTS) [22] on admission were extracted from the trauma registries. Data on comorbidity, scored using the age-adjusted Charlson comorbidity index [23, 24], and complications were collected from the medical records.

The primary outcome was in-hospital mortality. Secondary outcomes included hospital length of stay (HOS-LOS), intensive care unit admission and length of stay (ICU-LOS) ventilator-free days [25], readmission rates, complications (pneumonia, urinary tract infection (UTI), deep venous thrombosis (DVT), sepsis and wound infection) and discharge disposition.

### Statistical analysis

Demographic and clinical characteristics were compared by univariable analysis. Continuous variables were compared by the Pearson’s *t* test or Wilcoxon rank sum test, depending on data distribution. Categorical variables were compared by the Chi-squared test or Fisher’s exact test.

For in-hospital mortality, ICU admission, complications and (unplanned) readmission in NLTC compared to USTC, the odds ratio (OR) with 95% confidence interval (CI) were calculated using multivariable logistic regression analysis. Multiple linear regression analysis was used to calculate the mean difference (with 95% CI) in HOS-LOS and ICU-LOS between NLTC and USTC. Based on the literature and biological plausibility, potential clinically relevant confounders were analyzed. Age, gender, penetrating trauma mechanism, ISS and RTS were identified as clinically potential important confounders in the univariate analysis and adjusted for in all multivariable analyses. For this observational study, no hypothesis was pre-specified, and therefore, no formal sample size was calculated. Statistical testing was two-sided, and *p* values <0.05 were considered statistically significant. All statistical analyses were performed using IBM SPSS Statistics for Windows, version 23 (IBM Corp., Armonk, NY, USA).

## Results

### Comparison of trauma populations

**Fig. 1:**
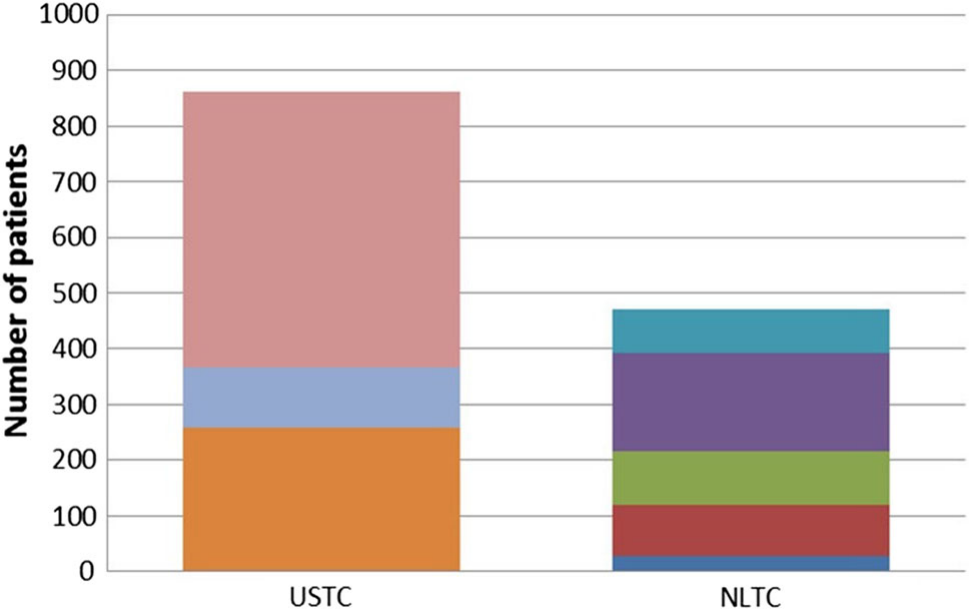
Number of patients with penetrating trauma, by trauma center location (USTC: 3 trauma centers in the USA; NLTC: 5 trauma centers in The Netherlands)

During the study period, 470 patients with truncal penetrating injuries were admitted in the NLTC and 861 in the USTC. The number of included patients per trauma center in each country is presented in Fig. 1. In general, more patients with penetrating trauma per trauma center were admitted in the USTC compared to the NLTC.

Table 1 summarizes the demographics and clinical characteristics in both centers. USTC patients were younger, slightly more often male and had a somewhat higher ISS than NLTC patients (median ISS 9 in both groups, *p* = 0.01), but no difference in RTS was seen. In USTC, significantly more patients with gunshot wounds were admitted (36.1 vs. 17.4%, *p* < 0.0001), which were more often the result of assault compared to NLTC. In both centers, the ISS of gunshot wound patients (NLTC median ISS 16 [interquartile range IQR 7.5–25] vs. USTC median ISS 16 [IQR 9–20], *p* = 0.82) was significantly higher compared to the ISS of patients with stab wounds (NLTC median ISS 9 [IQR 2–11] vs. USTC median ISS 6 [IQR 2–11], *p* = 0.64).

**Table 1:** Characteristics of patients with truncal penetrating injuries

	NLTC (*n* = 470)	USTC (*n* = 861)	*P*
Age [median (IQR)]	31.0 (24.0–34.5)	27.0 (22.0–37.0)	<0.0001
Male gender [*n* (%)]	410 (87.2)	783 (90.9)	0.03
Comorbidity [*n* (%)]	42 (9.2%)	88 (10.2)	0.30
Penetrating mechanism [*n* (%)]			
Stab wound	388 (82.6)	550 (63.9)	<0.0001
Gunshot wound	82 (17.4)	311 (36.1)	
Mechanism of injury [*n* (%)]			
Assault	365 (77.7)	785 (91.2)	<0.0001
Self-inflicted	82 (17.4)	52 (6.0)	
Other/unknown	23 (4.9)	24 (2.8)	
ISS, median (IQR)	9 (2–14)	9 (3–16)	0.01
RTS [*n* (%)]			
12	373 (86.3)	702 (86.1)	0.30
11	35 (8.1)	53 (6.5)	
<10	24 (5.6)	60 (7.4)	
GCS on admission [*n* (%)]			
GCS < 9	19 (4.4)	58 (6.8)	0.21
GCS 9–12	11 (2.6)	25 (2.9)	
GCS > 12	401 (93.0)	768 (90.1)	
SBP on admission [mean (SD)]	130.1 (27.6)	134.1 (31.3)	0.02
RR on admission [mean (SD)]	20.2 (11.2)	19.2 (5.0)	0.03
HR on admission [mean (SD)]	93.2 (22.3)	95.6 (26.1)	0.09

*NLTC* Netherlands trauma center, *USTC* US trauma center, *SD* standard deviation, *CCI* Charlson comorbidity index, *ISS* Injury Severity Score, *IQR* interquartile range, *RTS* Revised Trauma Score, *GCS* Glasgow Coma Scale, *SBP* systolic blood pressure in mmHg, *RR* respiratory rate per minute, *HR* heart rate in beats/min

**Fig. 2:**
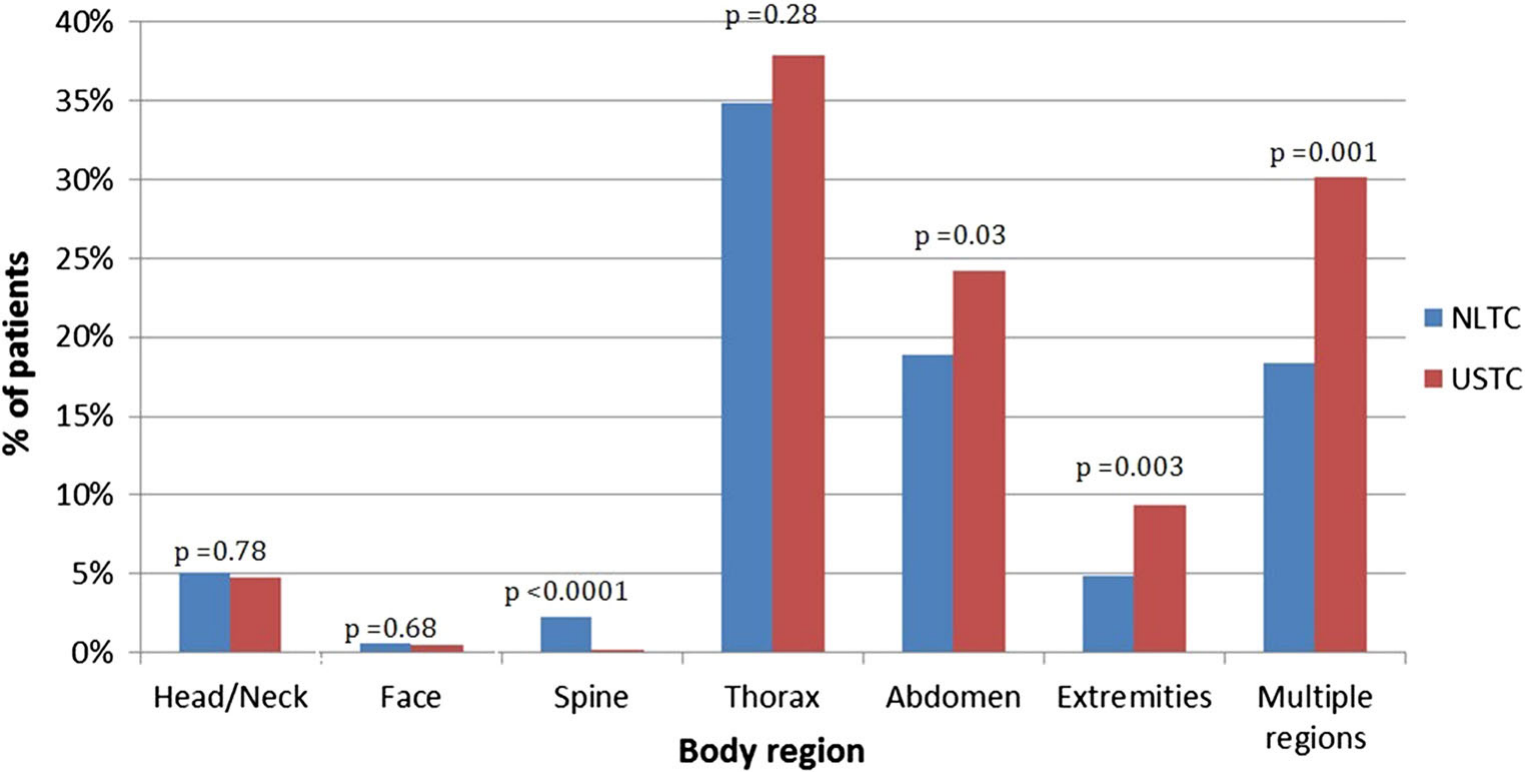
Percentage of patients with severe penetrating injury (AIS > 2), per body region by trauma center location

Figure 2 shows the distribution of severe penetrating injuries (AIS > 2) per body region in both centers. NLTC patients had more often severe injuries to the spine (2.3 vs. 0.1%, *p* < 0.0001), while in USTC patients more penetrating injuries to the abdomen (24.2 vs. 18.9%, *p* = 0.03), extremities (9.4 vs. 4.9%, *p* = 0.0003) and multiple body regions (30.2 vs. 18.4%, *p* = 0.001) were seen.

### In-hospital mortality

The in-hospital mortality rate in NLTC was 3.6% (17/470) compared to 5.0% (43/861) in USTC (*p* = 0.25) (Table 2). The unadjusted OR for mortality in the USTC compared to the NLTC was 1.40 (95% CI 0.75–2.49, *p* = 0.25). After correction for clinically relevant confounders, the adjusted OR for in-hospital mortality in the USTC compared to the NLTC was 0.95 (95% CI 0.35–2.54, *p* = 0.91). Higher ISS, RTS < 10 and gunshot wounds were statistically significant predictors of in-hospital mortality (Table 3). There was no difference in mortality in patients with gunshot wounds (NLTC 11.3% vs. USTC 11.0%, *p* = 0.48) and patients with stab wounds (NLTC 2.1% vs. USTC 1.5%, *p* = 0.94).

**Table 2:** Outcomes for patients with truncal penetrating injuries

	NLTC (*n* = 470)	USTC (*n* = 861)	*P*
In-hospital mortality [*n* (%)]	17 (3.6)	43 (5.0)	0.25
HLOS [median (IQR)]	3 (1–6)	2 (1–6)	0.11
ICU admission [*n* (%)]	134 (28.6)	291 (33.8)	0.05
ICU-LOS [median (IQR)]	1 (1–2)	2 (1–5)	<0.0001
Ventilator-free days^a^ [median (IQR)]	27 (26–28)	27 (25–28)	0.02
Mechanical ventilation^a^ [*n* (%)]	56 (47.1)	168 (58.1)	0.04
Complication [*n* (%)]			
Pneumonia	12 (2.6)	24 (2.8)	0.83
Urinary tract infection	4 (0.9)	15 (1.7)	0.20
Deep venous thrombosis	1 (0.2)	14 (1.6)	0.02
Sepsis	4 (0.9)	9 (1.0)	0.74
Wound infection	18 (3.9)	44 (5.1)	0.30
Discharge disposition [*n* (%)]			
Home	365 (80.6)	625 (86.9)	<0.0001
Mental health facility	42 (9.3)	40 (5.6)	
Rehabilitation	5 (1.1)	36 (5.0)	
Nursing home	11 (2.4)	5 (0.7)	
Other/unknown	30 (6.6)	13 (1.8)	
Readmission^b^ [*n* (%)]	17 (3.8)	46 (5.6)	0.15

*NLTC* Netherlands trauma center, *USTC* US trauma center, *HLOS* hospital length of stay in days, *IQR* interquartile range, *ICU* intensive care unit, *ICU-LOS* intensive care unit length of stay in days

^a^Of patients admitted to ICU

^b^Of patients surviving hospital admission

**Table 3:** Risk factors for in-hospital mortality in patients with truncal penetrating injuries

Risk factor	Odds ratio (95% CI)	*P*
Location		
NLTC	1 (reference)	
USTC	0.95 (0.35–2.54)	0.91
Gender		
Female	1 (reference)	
Male	0.60 (0.14–2.62)	0.49
Age	1.02 (0.99–1.06)	0.23
ISS	1.08 (1.04–1.13)	<0.0001
RTS		
12	1 (reference)	
11	4.28 (0.95–19.16)	0.06
<10	59.26 (20.62–170)	<0.0001
Type of trauma		
Stab wound	1 (reference)	
Shot wound	3.85 (1.37–10.81)	0.01

### Secondary outcome measures

HOS-LOS was similar in both centers (Table 2). After correction for differences in case mix, USTC HOS-LOS was on average 0.17 days shorter than in NLTC (95% CI −0.29 to −0.05, *p* = 0.005). A higher age, gunshot wounds, higher ISS and low RTS were statistically significant predictors of a longer LOS. The ICU admission rate in USTC appeared higher compared to NLTC (33.8 vs. 28.6%, *p* = 0.05), but this association was not statistically significant after adjustment for differences in case mix (OR 0.96, 95% CI 0.71–1.31, *p* = 0.80). ICU-LOS was significantly longer in USTC compared to NLTC (median 2 [IQR 1–5] days vs. 1 [IQR 1–2] days, *p* < 0.0001). This association remained statistically significant after correction for differences in case mix (difference of 0.39 days, 95% CI 0.18–0.60, *p* < 0.0001). A higher ISS and gunshot wounds were statistically significant predictors of a longer ICU-LOS. More ICU admitted USTC patients received mechanical ventilation than NLTC patients (47.1 vs. 58.1%, *p* = 0.04), after correction for clinically relevant parameters this difference was no longer statistically significant (OR 1.59, 95% CI 0.99–2.57, *p* = 0.06). DVT was more often diagnosed in USTC, and the incidence of other complications was similar in both countries (Table 2). This difference in DVT incidence ceased to exist after adjustment for differences in case mix (OR 3.0, 95% CI 0.36–35.1, *p* = 0.31).

A statistically significant difference in discharge disposition was seen (*p* < 0.0001), with more USTC patients being discharged to a rehabilitation center (5.0 vs. 1.1%), while more NLTC patients were discharged to a mental health facility (9.3 vs. 5.6%) or nursing home (2.4 vs. 0.7%). Readmission rates were similar, even after correction for differences in case mix (OR 1.4, 95% CI 0.75–2.71, *p* = 0.28) (Table 2).

## Discussion

In this binational collaboration between five Level-1 trauma centers in the Netherlands and three Level-1 trauma centers in the USA, we found that patient volumes, especially of gunshot victims, were significantly higher in the USTC compared to NLTC. Apart from patient volumes and trauma mechanism, the patient populations were fairly comparable with similar ISS and RTS. The in-hospital mortality was similar (4–5%) and comparable with or lower than rates reported in other studies [3, 26, 27].

Although the studied geographical areas in both countries had comparable urbanization and violent crime rates, the proportion of admitted patients with gunshot wounds was almost twice as high in the USTC. This is most likely due to differences in legislation concerning firearm use and ownership. Dutch citizens can only obtain a firearm permit under very strict conditions [28, 29], whereas guns can easily be obtained in the USA. Research has shown that a major determinant of firearm-related deaths is the availability of guns and that the implementation of restrictive laws in firearm purchase or access led to a reduction in firearm-related deaths in several countries, such as Australia, New Zealand, South Africa and Canada [30–32].

In our study, no difference in in-hospital mortality was found between both centers, despite that the penetrating trauma patient volumes in USTC were generally higher than in NLTC. Although it has been suggested that higher trauma patient volumes are associated with better outcomes, this relationship remains inconclusive due to heterogeneity of studies [33, 34]. Nevertheless, there is evidence that regionalization of trauma care may lead to reduced mortality rates [35]. Implementation of comprehensive trauma systems by regionalizing and standardizing complex trauma care in Level-1 facilities is likely to be more effective for improving outcomes after trauma than case volume itself [34, 36].

In both USTC and NLTC, all-inclusive trauma systems have been implemented that provide 24/7 acute trauma care and have similar facilities such as immediate availability of CT scanning, ICU beds and an in-house surgical team with an operating room available at all times. Surgeons and surgical residents in both systems receive similar surgical training, and management of penetrating trauma is broadly similar both following ATLS protocol [37]. Despite these similarities, some differences in clinical outcomes and processes were observed. Firstly, higher DVT rates were seen in USTC, although these differences ceased to exist after correction for differences in case mix. In both USTC and NLTC, patients received prophylactic treatment, mainly low molecular weight heparin, but inferior vena cava (IVC) filters were not routinely placed. Diagnostic approaches such as ultrasound were used if there were clinical signs indicating a potential DVT. Higher DVT rates might be explained by differences in clinical management; however, more likely it is explained by USTC patients experiencing more in the literature identified risk factors for DVT such as a younger age, and more thoracic and abdominal injuries [38, 39].

Secondly, although the ICU admission rates were similar, the ICU-LOS in USTC was somewhat longer. Although this might be explained by the larger numbers of patients with gunshot wounds with a higher ISS and of patients needing mechanical ventilation, the longer ICU-LOS in USTC is most likely due to the unavailability of floor beds which may delay ICU discharge, as the USTC operates at a constantly 100% capacity. Another likely explanation is the availability of medium care units in most of the NLTC, to which patients can be discharged when they are weaned from the ventilator but still need close monitoring. However, although statistically significant, the differences for both ICU—and HOS-LOS less than 1 day were too small to be considered clinically relevant.

Lastly, although the majority of patients in both groups were discharged home, there were noticeable differences in discharge protocol. Significantly more NLTC patients were discharged to a mental health facility possibly explained by the higher incidence of self-inflicted wounds in this population. More USTC patients were discharged to a rehabilitation center, possibly explained by the extensive network of rehabilitation centers in the USTC region with which they work closely. Despite these differences in hospital discharge policy, the readmission rates were similar.

### Strengths and limitations

The detailed data collection and the large cohort are strengths of our study. Data collected from the trauma registries were complemented by data from electronic medical records collected by one researcher (AH), limiting the amount of missing data. A limitation of our study was that no information on morbidity and mortality was available after hospital discharge. A second limitation is that we excluded specific patient groups from the study such as patients with isolated penetrating injuries to the brain and extremities. They are considered a different group, and the involvement of trauma surgery is often limited after the initial resuscitation phase. Additionally, all patients who were first managed in another hospital before being admitted to one of the participating centers were excluded. Although studies have shown that mortality is similar between transferred and non-transferred patients, differences in complication rates do exist [40]. By excluding these patients, we may have caused a selection bias in the study groups, since transfer rates were higher in USTC. However, it was not feasible to collect primary data for these patients, so we felt compelled to exclude them. A third limitation is that the study was performed in a limited number of trauma centers in both countries. Although we feel that the participating USTC and NLTC are representative for the Level-1 trauma centers in the densely populated urban areas in the USA and Netherlands, the results of this study may not allow for a comparison of care for patients with penetrating injuries in the two countries as a whole.

## Conclusion

Despite the higher incidence of penetrating trauma, particularly firearm-related injuries, and higher hospital volumes in the USTC compared to the NLTC in this study, the in-hospital mortality was similar in these centers. We also did not see clinically important differences in other outcomes between the centers in both countries. Despite variations in trauma system organization and clinical routine, implementation of all-inclusive trauma systems in both countries seems to have led to a comparable standard of care. More in-depth research is needed to uncover other potential factors that might contribute to differences in outcomes for specific patient subgroups, to further improve the care for penetrating trauma patients.
